# Measurement of perceived pressures in psychiatry: paper-and-pencil and computerized adaptive version of the P-PSY35 scale

**DOI:** 10.1186/s12991-024-00501-5

**Published:** 2024-05-10

**Authors:** Philippe Golay, Debora Martinez, Mizué Bachelard, Benedetta Silva, Alexandra Brodard, Jonathan Perrin, Nolan Pedro Fernando, Lou-Ann Renaud, Charles Bonsack, Stéphane Morandi

**Affiliations:** 1 Service of Community Psychiatry, Department of Psychiatry, Lausanne University Hospital and University of Lausanne, Consultations de Chauderon, Place Chauderon 18, 1003 Lausanne, Switzerland; 2 Service of General Psychiatry, Department of Psychiatry, Lausanne University Hospital and University of Lausanne, Lausanne, Switzerland; 3 Cantonal Medical Office, Directorate General for Health of Canton of Vaud, Department of Health and Social Action (DSAS), Avenue Des Casernes 2, 1014 Lausanne, Switzerland

**Keywords:** Informal coercion, Treatment pressures, Perceived coercion, Item response theory, Computerised adaptive testing

## Abstract

**Purpose:**

Formal coercion in psychiatry is widely studied yet much less is known about pressures patients may experience, partly because of the very few measures available. The goal of this study was to validate the P-PSY35 (Pressures in Psychiatry Scale) and provide a paper-and-pencil and a computerised adaptive test (CAT) to measure pressures experienced by patients in psychiatry.

**Methods:**

The P-PSY35 items were developed with users. Patients were evaluated during psychiatric hospitalisation or through an online survey. Mokken scale analysis and Item response theory (IRT) were used to select and estimate the items parameters. A Monte-Carlo simulation was performed to evaluate the number of items needed to transform the paper-and-pencil test into a reliable psychometric CAT.

**Results:**

A total of 274 patients were assessed. The P-PSY35 demonstrated good internal validity, internal consistency, convergent and divergent validity. The P-PSY35 could be substantially shortened while maintaining excellent reliability using the CAT procedure.

**Conclusion:**

The P-PSY35 was developed in collaboration with users. It is a psychometrically rigorous tool designed to measure experienced pressures in French-language. The development and successful validation of the P-PSY35 represent a welcome step towards implementing and evaluating programs aimed at reducing negative consequences of coercion.

## Introduction

In psychiatry, the use of coercion is justified by the need to protect the patients and/or other people. Formal coercion consists of the legal procedures to force someone into treatment while informal coercion comprises various forms of pressure used by medical staff or relatives to persuade someone to undergo treatment [1]. Perceived coercion describes the coercion experienced and felt by a person during treatment [2].

The negative impact of coercion has been well studied [3–7]. Formal coercion has been linked to a negative impact on patients’ quality of life and their clinical course [7]. Formal coercion was also associated with decreased satisfaction with care and treatment adherence in the long-term [3, 8]. Previous experience of formal coercion was linked to a higher risk of use of formal or informal coercion in the future [9–11]. Additionally, previous experience of coercive measures may impact patient satisfaction and increase their perception of coercion in subsequent voluntary hospitalisations [12]. Finally, disengagement from services and negative therapeutic relationships are also associated with perceived coercion [4, 6, 13].

While formal coercion in psychiatry has been comprehensively studied, there is a knowledge gap regarding the other forms of pressure experienced in psychiatry. In fact, much less is known about their specifics and potential short-, medium- and long-term adverse effects [14]. Because of their more subtle nature, several accounts of treatment pressures have been proposed in the literature. Lovell [15] described four forms of informal coercion that could be represented on a continuum [16, 17] from the most to the least coercive: coercion, coerced voluntarism, utilitarian compliance and persuasion. Lidz and colleagues [13] also proposed to distinguish positive and negative pressures within informal coercion. The key difference between these symbolic pressures lied in the willingness to encourage or threaten the person. Angell [18] developed a continuum of coercive strategies used by practitioners to maintain treatment compliance. This time, six forms of coercion were included: persuasion, monitoring, incentives, leverage, threat, and invocation of authorities.

The most widespread model of pressures in mental health literature is maybe that of Szmukler & Appelbaum [1]. Treatment pressures are presented as five ordered categories: persuasion, interpersonal leverage, inducements, threats and compulsion. As such, persuasion consists of appealing to the patient’s reason and emotions to make them accept a therapeutic measure. Interpersonal leverage consists of using the emotional connection caregivers or relatives have with the patient to get them to agree to a therapeutic measure. Inducements can be understood as making certain benefits (e.g., cigarettes) contingent on acceptance of a therapeutic measure (e.g., only if the patient takes medication). Threats can be described as suggesting to the patient that they will lose something (e.g., monetary or housing benefits) if they refuse a therapeutic measure. Lastly, compulsion is intended as legally forcing someone to undergo psychiatric treatment, by compulsorily admit them to hospital or commit them to undergo outpatient treatment (OPC). [1]. Among the pressures exerted on patients out of legal status, only threats were however identified by these authors as coercion [1].

Trying to clarify the terminology used in the literature, Yeeles defined “informal coercion” as “*a broad term covering various non- statutory treatment pressures used on a day- to- day basis by clinicians, carers, family members, and the welfare and criminal justice systems to improve patients’ stability and treatment adherence.*” [19]. This study focused on this specific form of pressures experimented by patients in the context of psychiatry and aimed to provide a rigorous psychometric tool to measure them. Indeed, nowadays it is difficult to measure treatment pressures with existing tools. In a recent literature review, we highlighted that several tools existed to assess the patients’ level of perceived coercion [15]. However, only specific steps of psychiatric care were usually covered, such as patients admission or their interactions with caregivers within the hospital. Few instruments were available to caregivers to evaluate their practice in other settings. The focus on the hospital setting is problematic because it leaves out a variety of contexts where pressures are used and experienced, as well as all forms of pressures applied by relatives. Indeed, results from a qualitative study indicate that patients experience feelings of disempowerment in daily life due to the close monitoring of their adherence to treatment by their informal caregivers [20]. Only one measure [21] included both in- and outpatient services. Burns and colleagues [22] proposed a 4-item instrument, adapted from Monahan and colleagues [23], that aimed, in the context of assisted outpatient treatment, to specifically measure patients’ experiences of leverage in four domains of the social welfare: finance, housing, criminal justice and child custody. However, these four items represented rather severe forms of informal coercion that are most often exerted by professionals but not by relatives.

In view of these observations, there is a need for a new tool able to provide an overview of the range of pressures that voluntary and involuntary patients may experience in various in- and outpatient settings. Therefore, the goal of this study was to develop and validate such a rigorous psychometric tool. The scale has been designed to assess the overall perception of pressure for a wide range of psychiatric disorder and, given its adaptive nature, to allow very short administration times. Moreover, the items content was designed to cover pressures from both professionals or relatives.

## Material and methods

### Participants

Participants were recruited between February 2022 and September 2023 using the following recruitment strategy: patients were recruited in six psychiatric hospitals in the French-speaking part of Switzerland, and through an online survey. The set of questions and scales was in both instances identical. Both hospital and online participants should be at least 18 years old and no older than 65 to be included in the study. People diagnosed with dementia (F00-F09) or Intellectual disability (F70-F79) were excluded. Moreover, participants from the online survey were informed that they could take part in the study only if they were or had been under psychiatric care, had a psychiatric diagnosis and were sufficiently proficient in French. A correct answer to two control items (i.e., “*In order to check your concentration, please answer "rather yes" to this question”*) and to have completed sociodemographic and diagnostic data were also required in order for online participants to be included in the analysis. In hospitals, participants were contacted by a research assistant (trained master degree psychology student) in the presence of their attending nurse who provided them information on the study. After a period of consideration, people who agreed to participate signed the consent form and were interviewed individually. The online survey was advertised on various social media platforms and was relayed by patients’ associations.

A total of 274 patients were recruited and included in the study, of which151 (55.1%) were women. Their age ranged from 18 to 64 years old (M = 37.86, SD = 12.70). Primary diagnosis, based on the International Statistical Classification of Diseases and Related Health Problems 10th Revision (ICD-10), were the following: Mental and behavioural disorders due to alcohol use (F10) N = 8 (2.9%), Mental and behavioural disorders due to psychoactive substance use (F11–F19) N = 11 (4.0%), Schizophrenia (F20–F29) N = 63 (23.0%), Mood affective disorders—mania (F30–F31) N = 29 (10.6%), Mood affective disorders – depression (F32–F39) N = 85 (31.0%), Neurotic, stress-related and somatoform disorders (F40–F48) N = 17 (6.2%), Personality disorders (F60–F69) N = 40 (14.6%), Psychological development disorders (F80–F89) N = 5 (1.8%) and No diagnostic information available (first psychiatric hospitalisation) N = 16 (5.8%).

### Measures

Patients were asked to report their gender, age, and most significant CIM-10 diagnosis. In some instances, patients were assessed during their first psychiatric hospitalisation and no diagnostic information was yet available.

#### Development of the pressures in psychiatry scale (P-PSY35)

Being interested in measuring the total amount of pressure experienced by patients, we aimed at designing a unidimensional scale including various forms and levels of severity of pressures, the Pressure in Psychiatry scale (P-PSY35). The items of our pressure questionnaire were generated based on a literature review and through several consultations with a peer specialist and an expert panel [24]. The objective was to generate many items in order to select the best subset for the final scale. Because we were interested in the possibility of measuring change between different measurement occasions, we instructed patients to answer based on the last 3 months period. If needed, this instruction can be easily modified to assess the lifetime experience of treatment pressures in psychiatry.

A research assistant with lived experience of mental illness and recovery embedded within the research team and two psychologists trained in psychometrics and questionnaire development conducted the literature review. About 10 domains in all aspects of life (e.g., health, therapeutic means, belief, finance, work, education, social activities, addiction) related to pressures (stay well pressures, monitoring, persuasion, interpersonal pressure, leverage, threats, deception, decision of one another, show of force, use of violence) were identified. These domains served as a guide to generate items. The peer specialist was involved in reviewing the items suggested by psychologists and proposing new ones. In total, about 200 items were identified. After removing potential duplicates and ill-formulated items, this set was reduced to roughly 115 items. The items were further reviewed and selected using an expert panel session to improve content validity. The panel included three mental health professionals with a track record of research on coercion. All items were reviewed one by one, and changes were discussed on a consensus basis: The first step was to ask panel experts to read all items. The second step involved discarding, rephrasing, or suggesting new items. Items were modified one at a time directly on the screen during the open discussion until validation by all the participants [24]. The final questionnaire contained 98 items answered on a 5-point Likert scale: 0 = *“Not at all”*, 1 = *“Not much”*, 2 = *“Neutral”*, 3 = *“A little bit”*, and 4 = *“Definitively”*.

#### Coercion ladder

The Coercion Ladder [25] was originally adapted from the Cantril Ladder [26]. It is a visual analogue tool on which the patient is asked to mark the degree of perceived coercion on a scale from 1 (Minimum use of coercion) to 10 (Maximum use of coercion). Participants were instructed to answer in relation to their entire experience of psychiatric care.

#### Coercion experience scale (CES)

The CES [27] is a scale designed to measure patients’ experiences of coercive measures. The scale was first developed in German before being translated and published in English [27] and then validated in French [28]. In this study, we only used the second item which has been designed to evaluate the extent to which patients consider coercive measures stressful on a visual analogue scale from 0 to 100.

#### Informal coercion dichotomous items

Pressures to adhere to treatment (‘leverage’) were assessed using a 4-item instrument proposed by Burns and colleagues [22], which was adapted from Monahan and colleagues [23]. It aims to measure patients’ lifespan experiences of leverage in four domains of the social welfare: finance, housing, criminal justice and child custody. These items represent rather severe forms of informal coercion. They correspond to inducements and threats as defined by Szmukler & Appelbaum’s [1].

#### Satisfaction regarding hospitalisation (ANQ)

The Swiss National Association for Quality Development in Hospitals and Clinics (ANQ) developed a satisfaction measure for patients in psychiatry. The questionnaire includes 6 five-point Likert-type items assessing quality of treatment, information and communication, medication, patient’s implication and discharge preparation [29]. We used the first item (that focused on the perceived quality of psychiatric care) and the total score (that can be computed to assess the global satisfaction of the patient).

#### The self-stigma scale—short (SSS-S)

The SSS-S is a 9-item questionnaire designed to measure the degree of self-stigma of individuals from various minority groups. It consists of a cognition score, an affect score, a behaviour score, and a total score. In the present study, we used the French-version of the SSS-S [30].

#### The rosenberg self-esteem scale (RSS)

The RSS is the most frequently used instrument to measure self-esteem [31]. It consists of 10 items with a total score ranging from a minimum of 10 to a maximum of 40. Participants respond on a Likert scale by checking one of the four options: *“strongly disagree”*, *“disagree”*, *“agree”*, and *“strongly agree”*.

#### The beck hopelessness scale (BHS)

The BHS is a widely used questionnaire that measures negative expectations about the future [32]. The inventory is a self-report measure and consists of 20 items scored on a true–false scale. A total score can be computed and ranges from 0 to 20, with higher scores reflecting higher levels of hopelessness. In the present study, we used the French-version of the BHS [33].

#### Self-reported health

One item of the ANQ questionnaire is a self-reported five-point Likert-type item about the patient self-perceived global health [29]. Patients can rate their perceived actual health between “*bad*” and “*excellent*”.

### Procedure

The internal validity of the P-PSY35 was assessed first. This phase aimed to select the final set of items on the basis of the internal structure of the test. The reliability of the scale and the model goodness of fit were then estimated. Next, to evaluate convergent and divergent validity, we studied the relationship between the P-PSY35 score and several other scales. We hypothesised that the P-PSY35 scores would be positively correlated with the Coercion ladder, the CES 0–100 item, the Informal coercion dichotomous items, the SSS-S and the BHS scores. We also hypothesized a negative correlation with the ANQ and the RSS scores. To evaluate the divergent validity, we hypothesized we would find no significant correlation between the P-PSY35 and the Self-reported Health measure.

Finally, a Monte-Carlo simulation was performed to evaluate the number of items needed to transform the paper-and-pencil test into a psychometric CAT with a high reliability (r ≥ 0.90).

### Statistical analysis

#### Internal validity

Given the large number of items at the beginning of the procedure, these were first screened using Mokken scale analysis. This is a non-parametric method based on the monotonicity of the item response function. Items with low scalability (Ho < 0.30) were discarded. The “mokken” R-package was used [34]. Next, remaining items were selected on the basis of an item fit statistic. We discarded items with significant signed chi-squared test [35, 36]. The Multidimensional Item Response Theory (mirt) package for R was used [37]. Finally, items pairs were screened for local dependency using Yen's Q3, with values under 0.3 suppressed [38, 39]. From the locally dependent pairs, we deleted items which had less information based on their information curves. A final item fit statistic test was performed at the end of the item selection procedure to ensure all final items did not contribute negatively to the overall fit of the scale. Model fit and items parameters were then estimated using the mirt package for R and a graded response model. Several indicators of model fit were used: the Root Mean Square Error of Approximation (RMSEA), the Tucker–Lewis fit Index (TLI), the Comparison Fit Index (CFI) and the Standardized Root Mean Squared Residual (SRMSR). RMSEA values ≤ 0.06, CFI and TLI values ≥ 0.95, and SRMRS ≤ 0.08 were interpreted as good fits, whereas RMSEA values ≤ 0.08, CFI and TLI values ≥ 0.90 and SRMRS ≤ 0.10 were considered as indicating acceptable fit [40].

#### Reliability

The reliability of the P-PSY35 scale was estimated using McDonald’s model-based Omega (ω) [41] and Cronbach’s alpha (α) coefficients. We also estimated the Marginal reliability (Rxx) [42]. Reliability coefficients above 0.80 were considered good and above 0.90 were considered excellent [41, 43]. The “psych” and “mirt” R-package were used [37, 44].

#### Convergent validity

The convergent validity coefficients between the P-PSY35 and the other scales were estimated using Pearson correlation coefficients and Spearman’s Rho coefficient when the indicator was dichotomous. Under Classical Test Theory (CTT) the score reliabilities (more precisely their square root) act as an upper bound for validity coefficients. Therefore, the acceptable range is typically lower than for reliability coefficients [45]. Correlation coefficients between 0.40 and 0.60 were considered as good and any values higher than 0.30 (a medium effect size, according to Cohen [46]) as satisfactory.

#### CAT Simulations

We used the Firestar software designed to simulate CAT with polytomous items [47]. A large number of participants (10,000) were simulated to achieve accurate estimates under reasonable computing time. The simulated thetas were sampled from a normal distribution with a mean of 0 and a standard deviation of 1 which correspond to the level and dispersion of the original sample. Minimum and maximum thetas ranged between − 4 and 4 with increments of 0.05. The maximum number of items to administer was set to 35 and the minimum was 2. The stopping rule was set to a standard error corresponding to a reliability of 0.90. Interim theta estimations were carried out using expected a posteriori (EAP) estimations. The next items were selected using the Fisher maximum information method. During the Firestar simulation, we recorded the minimum, maximum, mean and median numbers of items administered before the stopping criterion. Pearson correlations were estimated between simulated and estimated thetas, and the mean reliability was based on the final standard errors.

## Results

Out of the 98 original items, results of the Mokken analysis allowed us to discard 17 items (#2, #20, #21, #22, #23, #30, #37, #39, #44, #54, #56, #60, #64, #66, #70, #91 & #95). The 81 remaining items were then subjected to item fit analysis. It allowed us to discard 24 items (#3, #5, #11, #13, #15, #16, #31, #32, #33, #38, #42, #43, #47, #50, #57, #63, #69, #73, #80, #87, #89, #90, #92, #94). The 57 remaining items were subjected to local independence analysis. Examination of items pairs allowed use to discard 20 items (#4, #7, #8, #17, #18, #24, #27, #28, #29, #45, #55, #58, #61, #65, #68, #76, #81, #82, #88, #97). Finally, two additional items were discarded based on a significant signed chi-squared test (#36, #86). The final model was fitted on the remaining 35 items (Table 1). The English language translation of the final items is provided in Table 2.

**Table 1:** French language version of the P-PSY35

Item—Durant les 3 derniers mois, en raison de mes problèmes de santé psychique:	
1	On a fait dépendre certains avantages personnels de ma collaboration au traitement psychiatrique
2	On m'a forcé·e à participer à certaines activités thérapeutiques
3	Mes proches ou des soignant·e·s ont appelé la police ou la sécurité pour que je sois hospitalisé·e ou que je suive un traitement psychiatrique
4	Des soignant·e·s ou des proches m'ont rappelé de suivre le traitement recommandé
5	On m'a menacé·e d'être mis·e sous tutelle ou curatelle si je ne suivais pas le traitement psychiatrique recommandé
6	Mes proches ou mes soignant·e·s m'ont menacé·e de ne plus m'offrir leur soutien si je ne changeais pas ma manière d'être ou ma vision du monde
7	On a demandé à mes proches de veiller à ce que je suive le traitement recommandé
8	On m'a fait comprendre que je n'avais pas d'autres choix que de suivre le traitement psychiatrique proposé
9	J'ai été encouragé·e à changer ma manière d'être et ma vision du monde
10	On m'a fait croire qu'il n'y avait pas d'autres alternatives que de suivre un traitement psychiatrique pour obtenir de l'aide pour mes affaires personnelles
11	Les professionnel·le·s de la santé m'ont caché des informations qui ne s'alignaient pas avec leur projet thérapeutique
12	Mes proches ou des soignant·e·s m'ont fait du chantage affectif pour me décider à suivre un traitement psychiatrique
13	Durant le suivi, on a fait dépendre la levée de certaines mesures de ma décision de suivre un traitement
14	Des soignant·e·s ont exprimé de la déception ou de la colère concernant mes choix thérapeutiques
15	Durant le suivi, on a fait dépendre certains avantages comme des visites, un congé ou de l'argent de poche de ma décision de suivre un traitement
16	Mes proches m'ont supplié·e de suivre le traitement psychiatrique recommandé
17	On m'a menacé·e de problèmes juridiques si je ne suivais pas le traitement
18	On m'a menacé·e de perdre l'accès à certains soins si je ne réduisais pas ma consommation d'alcool ou de drogue
19	Des soignant·e·s ou des proches ont contrôlé si je suivais le traitement recommandé
20	Mes proches m'ont menacé·e physiquement pour que je suive un traitement psychiatrique
21	Mes proches m'ont emmené·e de force à l'hôpital ou pour me faire suivre un traitement psychiatrique contre mon gré
22	Mes soignant·e·s sont venu·e·s à plusieurs pour m'ordonner de suivre un traitement psychiatrique
23	On m'a prédit une déroute financière si je ne suivais pas le traitement psychiatrique recommandé
24	Mes proches ou mes soignant·e·s ont fait dépendre leur soutien d'un changement de ma manière d'être ou de ma vision du monde
25	Durant le suivi, on m'a dit que suivre un traitement était une condition pour pouvoir sortir de l'hôpital/sortir de la chambre de soins/chambre d'isolement
26	Mes proches m'ont contraint·e physiquement de suivre un traitement psychiatrique
27	Mes soignant·e·s ont eu des comportements d'intimidation à mon égard pour que je suive un traitement psychiatrique
28	Des soignant·e·s ont dit qu'ils ou elles seraient déçu·e·s, tristes ou fâché·e·s si je refusais de suivre le traitement recommandé
29	Mes proches ont fait pression sur mes soignant·e·s concernant mes choix thérapeutiques
30	On a ignoré mes choix thérapeutiques
31	On m'a menacé·e de m'enlever des aides financières si je ne suivais pas le traitement psychiatrique recommandé
32	On m'a dit que si je ne suivais pas mon traitement je serais forcé·e d'aller à l'hôpital
33	Mes proches m'ont menacé·e de prévenir les autorités ou le médecin si je refusais le traitement psychiatrique recommandé
34	Plusieurs personnes m'ont fortement suggéré d'être aidé·e pour gérer mes affaires personnelles
35	On m'a persuadé que suivre un traitement pourrait fortement améliorer ma situation personnelle

Instructions: Ce questionnaire vise à évaluer les pressions que vous pourriez subir en raison de problèmes de santé mentale, et qui pourraient limiter votre liberté de choix ou votre autonomie. On parle de toutes les incitations, persuasions, pressions, menaces ou chantages exercés à votre égard pour vous faire accepter des mesures thérapeutiques. La pression peut être exercée par vos proches, soignant·e·s, curateur·trice, tuteur·trice etc. et dans tous les domaines de votre vie: bien-être, vision du monde, finances, aides sociales, logement, justice, garde des enfants, soins, consommations d’alcool, de médicaments non prescrits ou de substances, emploi, formation, activités sociales et loisirs, etc. Il ne s’agit pas de contrainte dite « formelle» pour lesquelles une décision légale a été prise (par exemple une hospitalisation non volontaire). Ces questions concernent ces trois derniers mois mais pas d’éventuelles expériences antérieures. Certains items pourraient vous sembler très similaires les uns des autres mais ils ne sont jamais identiques et décrivent des situations légèrement différentes

Options de réponse: 0 = *Pas du tout*; 1 = *Plutôt non*; 2 = *Neutre*; 3 = *Plutôt oui*; 4 = *Tout à fait*

**Table 2:** English language version of the P-PSY35

Item—During the last 3 months, because of my mental health problems:	
1	Some of my personal privileges were made contingent on my collaboration with psychiatric treatment
2	I was forced to take part in various therapeutic activities
3	My loved ones or professional caregivers called the police or security to have me hospitalised or undergo psychiatric treatment
4	My professional caregivers or loved ones reminded me to follow my recommended treatment
5	I was threatened with being placed under guardianship if I failed to follow my recommended psychiatric treatment
6	My loved ones or professional caregivers threatened to stop providing me with their support if I did not change my behaviour or my way of looking at things
7	My loved ones were asked to ensure that I followed my recommended treatment
8	I was made to understand that I had no choice but to follow the proposed psychiatric treatment
9	I was encouraged to change my behaviour and my way of looking at things
10	I was led to believe that there were no alternatives to following psychiatric treatment if I wanted to get help with my personal affairs
11	The healthcare professionals hid information from me that did not align with their therapeutic plans
12	My loved ones or professional caregivers emotionally blackmailed me into deciding to undergo psychiatric treatment
13	During the follow-up, the lifting of certain measures was made contingent on my decision to undergo treatment
14	Some professional caregivers expressed disappointment or anger concerning my treatment choices
15	During the follow-up, some of my privileges — such as visits from relatives, visits home or pocket money — were made contingent on my decision to continue my treatment
16	My loved ones begged me to follow the recommended psychiatric treatment
17	I was threatened with legal problems if I did not follow my treatment
18	I was threatened with losing access to certain types of care if I did not reduce my consumption of alcohol or drugs
19	Professional caregivers or loved ones monitored whether I was following the recommended treatment
20	My loved ones physically threatened me so that I would undergo psychiatric treatment
21	My loved one brought me to hospital by force or forced me to undergo psychiatric treatment against my will
22	A number of my professional caregivers came to see me at the same time to order me to undergo psychiatric treatment
23	I was told that I would face financial ruin if I did not follow the recommended psychiatric treatment
24	My loved ones or my professional caregivers made their support contingent on a change in my behaviour or my way of looking at things
25	During the follow-up, I was told that undergoing treatment was a precondition for being able to leave the hospital or the seclusion room
26	My loved ones physically forced me to undergo psychiatric treatment
27	My professional caregivers used intimidating behaviour against me so that I would undergo psychiatric treatment
28	My professional caregivers said that they would be disappointed, sad or angry if I refused to undergo the recommended treatment
29	My loved ones put pressure on my professional caregivers concerning my therapeutic choices
30	My therapeutic choices were ignored
31	I was threatened with the withdrawal of my financial aid if I did not undergo the recommended psychiatric treatment
32	I was told that I would be forced to go to hospital if I did not follow my treatment
33	My loved ones threatened to alert the authorities or my physician if I refused the recommended psychiatric treatment
34	Several people strongly suggested that I get help managing my personal affairs
35	I was persuaded that undergoing treatment would significantly improve my personal circumstances

Instructions: This questionnaire aims to evaluate the pressures that you might be subjected to because of your mental health problems and that might limit your freedom of choice or autonomy. We are talking about any incentives, persuasion, pressure, threats or blackmail that you might be subjected to in order to make you accept therapeutic mental health measures. These pressures might be exerted on you by your loved ones, family, relatives or close friends, your professional caregivers, or your legal guardian or trustee. They could apply to any area of your life, including your well-being, your way of looking at things, your finances, social benefits or legal situation, childcare, healthcare, your consumption of alcohol, non-prescribed medication or illicit substances, your job, training courses, social and leisure activities, and so on. The statements do not apply to official restrictions imposed upon you following a legal decision (for example, non-voluntary hospitalisation). The following statements for you to respond to relate to the last 3 months and not to any potential previous experiences. Some of the statements might seem very similar, but they are never identical and always describe slightly different circumstances

Response options: 0 = *Not at all*; 1 = *Not much*; 2 = *Neutral*; 3 = *A little bit*; 4 = *Definitely*

**Fig. 1:**
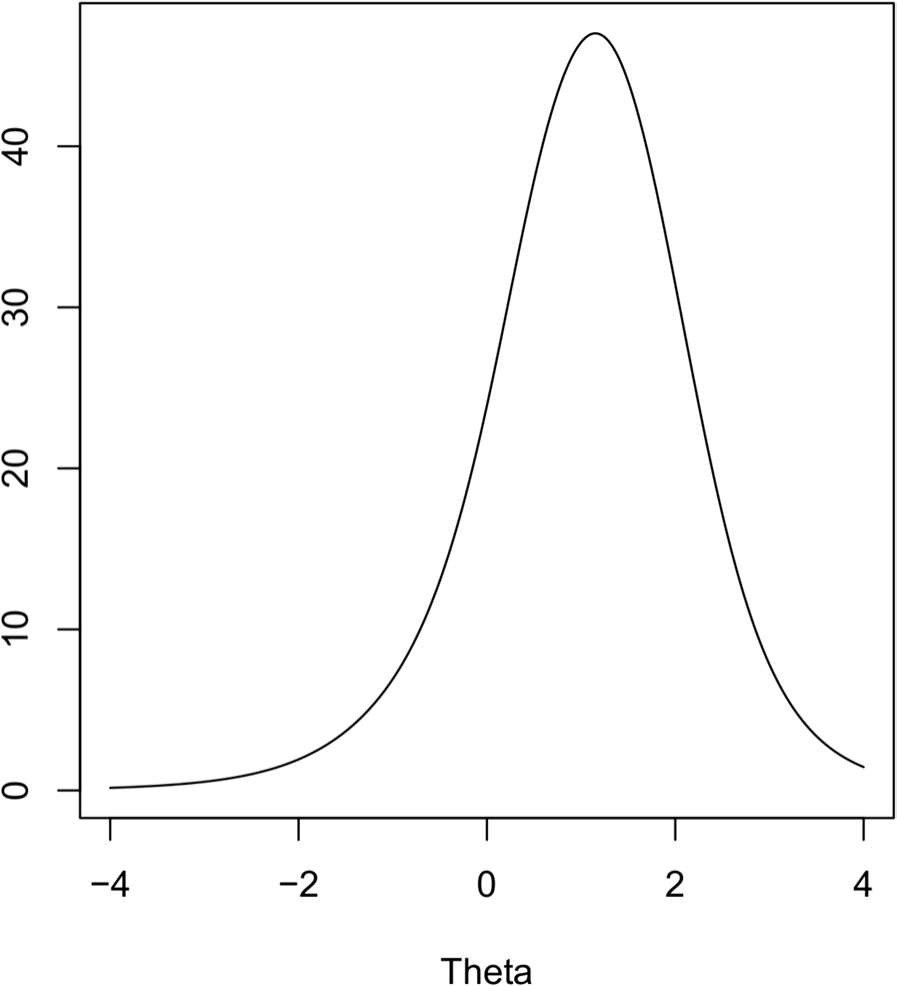
Information curve of the P-PSY35-

Estimate of model goodness of fit indicated adequate overall model fit (RMSEA = 0.0648; TLI = 0.9065; CFI = 0.9132; SRMR = 0.0806). The P-PSY35 total information curve is presented in Fig. 1. The maximum information is reached when theta equals 1.15 standard deviation above the mean. Items loadings and parameters are provided in Table 3. All 35 items had substantial loadings.

**Table 3:** Final scale items loadings and graded response model item parameters

Final item number	Original item number	Loadings	a	b1	b2	b3	b4
1	1	0.590	1.2428	− 0.2362	0.2741	0.7225	1.6286
2	6	0.656	1.4806	0.6616	1.2279	1.5109	2.3312
3	9	0.694	1.6422	0.6251	0.7045	0.8162	1.1519
4	10	0.551	1.1242	− 0.8891	− 0.5004	− 0.1801	0.8856
5	12	0.806	2.3190	0.9574	1.2133	1.3638	1.7630
6	14	0.719	1.7618	0.6620	0.9368	1.1644	1.8328
7	19	0.705	1.6897	0.4323	0.7104	1.0370	1.6056
8	25	0.729	1.8111	− 0.6386	− 0.3005	− 0.0003	0.7084
9	26	0.457	0.8740	− 1.0814	− 0.7647	− 0.2256	1.4743
10	34	0.760	1.9883	0.2496	0.5752	0.8763	1.4166
11	35	0.730	1.8189	0.3830	0.6886	1.1691	1.8094
12	40	0.811	2.3602	0.5746	0.7474	0.9682	1.4151
13	41	0.898	3.4809	0.5455	0.7280	0.9575	1.2758
14	46	0.784	2.1505	0.4911	0.8302	1.1379	1.7429
15	48	0.770	2.0563	0.8611	1.0787	1.3142	1.7147
16	49	0.719	1.7610	0.3564	0.5817	0.8895	1.4853
17	51	0.853	2.7799	0.9629	1.1124	1.2518	1.5557
18	52	0.679	1.5759	1.5087	1.7078	2.0439	2.4524
19	53	0.579	1.2078	− 0.4671	− 0.3030	− 0.0793	0.9122
20	59	0.868	2.9696	1.3420	1.5884	1.8149	2.1679
21	62	0.840	2.6390	0.7206	0.9353	1.0655	1.5066
22	67	0.785	2.1533	0.5990	0.8846	1.1000	1.4387
23	71	0.742	1.8866	1.1920	1.3423	1.6635	2.3315
24	72	0.769	2.0491	0.2581	0.4112	0.6631	1.4599
25	74	0.829	2.5275	0.2357	0.3239	0.5475	0.8757
26	75	0.846	2.7031	1.2511	1.4246	1.7344	1.9122
27	77	0.814	2.3856	0.6500	0.8658	1.0795	1.5491
28	78	0.772	2.0639	0.7632	0.9805	1.3125	2.1297
29	79	0.763	2.0088	0.7095	1.0984	1.3921	1.8676
30	83	0.745	1.9008	− 0.0094	0.4288	0.7521	1.2817
31	84	0.876	3.0944	1.0930	1.2659	1.5312	1.7328
32	85	0.824	2.4720	0.4136	0.6532	0.8745	1.1331
33	93	0.861	2.8765	0.8103	0.9778	1.2483	1.6092
34	96	0.660	1.4941	0.1812	0.3863	0.6229	1.4231
35	98	0.704	1.6870	− 0.8698	− 0.5104	− 0.2047	0.5410

Estimates of reliability were excellent (ω = 0.950; α = 0.949; Marginal reliability Rxx = 0.925).

Correlations between the P-PSY35 and other scales are presented in Table 4. Most correlation coefficients were substantial, significant and in the expected direction indicating good convergent validity. Correlations between the P-PSY35, the RSS and the BHS were typically lower and not statistically significant. To elucidate whether this could be attributed to the P-PSY35 scale or if it depicted a more general result indicating no relationship between pressures and Self-esteem respectively Hopelessness, we conducted a post-hoc analysis. We correlated the informal coercion dichotomous items (Finance, Housing, Criminal Justice & Child Custody), the RSS and BHS scores. Correlation ranged from − 0.015 to 0.115 for the RSS score, and from − 0.061 to − 0.147 for the BHS score respectively. This indicated that informal coercion measured by other means than the P-PSY35 was also not related to Self-esteem or Hopelessness. Estimates of divergent validity between the P-PSY35 and Self-reported health indicated that pressures were, as expected, not related to perceived health.

**Table 4:** Convergent and divergent validity of the P-PSY35 score

	P-PSY35 total score
Convergent validity	
Coercion ladder	0.488*
Perception of coercion as stressful 0–100 item (CES)	0.521*
Informal coercion dichotomous items	
Finance	0.226*
Housing	0.465*
Criminal justice	0.352*
Child custody	0.125*
Satisfaction (ANQ)	
Item 1 (perceived quality of psychiatric care)	− 0.270*
Total satisfaction score	− 0.415*
Self-Stigma (SSS-S)	
Cognitive score	0.272*
Affective score	0.240*
Behavioral score	0.107
Total score	0.243*
Self-esteem (RSS)	0.074
Hopelessness (BHS)	− 0.007
Divergent validity	
Self-reported health	0.032

^*^p < .05

Based on the 35 items’ bank, a mean of 15.256 items (SD = 11.671) was administered for the P-PSY35 scale using CAT, with the number of items needed to achieve the expected reliability varying between 3 and 35. The median number of items was 10. Average reliability was 0.891, and the correlation between the simulated and estimated thetas was close to unity (r = 0.978).

Finally, to facilitate clinical use, normative data on the total sample are presented in Table 5. The adaptive version of the P-PSY35 is also directly and freely accessible online for clinicians or researchers [48].

**Table 5:** Normative data for the paper and pencil version of the P-PSY35

	Stanine 1	Stanine 2	Stanine 3	Stanine 4	Stanine 5	Stanine 6	Stanine 7	Stanine 8	Stanine 9
%	4.0%	6.6%	12.1%	17.5%	19.6%	17.5%	12.1%	6.6%	4.0%
	Very low	Low		Average			High		Very high
P-PSY35 total score	0–1	2–4	5–12	13–24	25–38	39–68	69–86	87–98	99–140

## Discussion

The aim of this study was to develop in close collaboration with users and validate an instrument measuring pressures experienced in psychiatry in French language. The items were generated based on a literature review and the collaboration with people with mental health problems and experts on coercion. The P-PSY35 proved to be a reliable and valid instrument which measures pressures in psychiatry. The P-PSY35 demonstrated good internal validity, internal consistency, convergent and divergent validity on a varied psychiatric sample. The P-PSY35 could be substantially shortened while maintaining excellent reliability using the CAT procedure.

The final scale demonstrated a good model fit and a high reliability of the test scores. Much shorter measures with excellent reliability could also be obtained using CAT. Patients may be tired or may find questionnaires too long. When the time required to complete a psychometric questionnaire constitutes a barrier to effective clinical evaluation, professionals should have access to shorter but equally accurate tests. Today, open-source, online adaptive testing platforms, such as Concerto, are freely available [49]. Efforts to modernise test engineering using computerised adaptive testing (CAT) models make it possible to increase the comfort of testing for patients without altering data quality.

Good convergent validity was evidenced with significant relationship between P-PSY35 scores and global measures of experienced coercion and more specific informal coercion measures. As hypothesized, P-PSY35 scores were negatively correlated with satisfaction with psychiatric care. While our measure of pressures was positively correlated with cognitive and affective measures of Self-Stigma, we did not find a relationship with behavioural aspects of self-stigma. We may hypothesize that pressures experienced in psychiatry may have an impact on affect and cognition, yet no substantial effects on behaviours. This is interesting considering that theories of coercion define coercion as mainly a behavioural phenomenon [50].

Interestingly, we also did not find a relationship between pressures, Self-esteem and Hopelessness. This finding may be robust and not limited to the P-PSY35 because informal coercion measured by other means than the P-PSY35 was also not related to Self-esteem or Hopelessness. This highlights the need of measuring pressures more specifically, with a new scale such as P-PSY35. This may be related to the notion of paradoxical empowerment [51–53]. Within the concept of self-stigma and its well-documented negative consequences, research has also outlined a paradox: some people react to stigma by being righteously angry and becoming more empowered to fight against the injustice experienced [52, 54–56]. Righteous anger and coming out proud might therefore protect people from detrimental effects of stigma and this phenomenon may contribute to explain why Self-esteem and Hopelessness were not affected by experienced pressures.

Our study has several limitations that could be the focus of future research. First, our study did not consider diagnostics but aimed at covering a wide range of psychiatric conditions. Second, this study was mainly cross-sectional and longitudinal designs may be used to examine the P-PSY35 sensitivity to change. Third, even if our sample of 274 participants is substantial, further studies may be useful to replicate our findings on bigger samples. Fourth, our item generation process did not include a systematic rating of items by the participants. Therefore, content validity indexes could not be calculated. Fifth, we acknowledge that pressures felt by patients could be a byproduct of various factors such as treatment or aggressiveness. Sixth, even if all patients were informed that the investigators were independent of the hospital staff and that their responses would not be transmitted to anyone, we cannot exclude the possibility of response distortion. Seventh, we examined the potential for scale length reduction using a simulation approach as it is the only way to assess whether our instrument would be able to accurately recover the participants’ theta value: this value is known in the simulation context but totally unknown with real participants. While simulation work may have excellent internal validity, it may lack external validity. Finally, because of the convenience sampling procedure, refusals or response rate were not documented.

The significance of our results lies in the additional possibility offered to study various aspects of pressures experienced in psychiatry in French-speaking populations. Moreover, pressures were not related to perceived health status. Therefore, the P-PSY35 may not be particularly biased with patients with very low perceived health. We hope this tool will allow us a better understanding of coercion and its effects, to monitor and evaluate programs aimed at reducing its negative consequences and to have a significant impact on treatment.

Regarding individual actions, mental health professionals should be encouraged to discuss the topic and implications of pressures with their patients. The P-PSY35 could be an effective tool to monitor different aspects of coercion but also to stimulate discussion around this topic with everyone involved in treatment.

Regarding community responsibilities, the negative consequences of coercion and the need for specific interventions must be put at the top of the agenda. Awareness campaigns must be developed to ultimately reduce coercion with health professionals but also with relatives. Regarding policy implication, additional regulations are obviously needed to protect patients from coercion and warrant them access to specialized care and adequate treatment. Pressures can be exerted by professionals or relatives in order to improve treatment adherence or to limit the use of formal coercion [13, 14, 57] but the perception of not being involved in a fair decision making process (procedural justice) can reinforce perceived coercion with detrimental effects [58, 59].

## Conclusion

Coercion is still too often associated exclusively with formal measures such as involuntary hospitalisation, seclusion or restraint. Having tools to measure pressures makes it possible to highlight the more insidious forms of coercion faced by people suffering from mental disorders, to make patients and those around them aware of these pressures and to consider approaches aimed at limiting their use, given their potential negative effects on the people concerned. The P-PSY35 is a psychometrically rigorous tool developed in close collaboration with users and designed to measure pressures experienced in psychiatry in French. The development and validation of the P-PSY35 represent a welcome step towards implementing and evaluating programs aimed at reducing negative consequences of coercion.

## Data Availability

The data that support the findings of this study are available from the corresponding author upon reasonable request.
